# Photodynamic detection of diseased axillary sentinel lymph node after oral application of aminolevulinic acid in patients with breast cancer

**DOI:** 10.1038/sj.bjc.6601615

**Published:** 2004-02-17

**Authors:** K A Frei, H M Bonel, H Frick, H Walt, R A Steiner

**Affiliations:** 1 Department of Obstetrics and Gynaecology, University Hospital Bern, Inselspital, Effingerstrasse 102, Bern CH-3010, Switzerland; 2 Department of Radiology, University Hospital Bern, Inselspital, Bern CH-3010, Switzerland; 3 Department of Pathology, Cantonal Hospital Chur, Chur 7000, Switzerland; 4 Research Division of Gynaecology, Department of Obstetrics and Gynaecology, University Hospital Zurich, Zurich 8091, Switzerland; 5 Kantonal Women's Hospital Fontana, Luerlibadstrasse 118, Chur 7000, Switzerland

**Keywords:** aminolevulinic acid, photodynamic diagnosis, breast cancer, sentinel lymph node, axillary lymph node

## Abstract

Benign as well as malignant tumour tissues of the breast demonstrate higher fluorescence intensity (FI) than normal breast tissue after application of a photosensitiser. As a follow-up study, we evaluated the FI of metastatic sentinel lymph nodes and metastatic axillary lymph nodes compared to nonmetastatic sentinel and axillary lymph nodes in patients with breast cancer. In all, 11 patients received 30 mg 5-aminolevulinic acid (ALA) kg^−1^ bodyweight orally 3 h prior to surgery. The sentinel lymph node was marked with Nanocoll® preoperatively and with a blue dye intraoperatively. Tumour excision, excision of the sentinel lymph node and an axillary lymph node dissection were performed during the same surgical session. The operation site was illuminated with blue light (400 nm) to obtain macroscopic tissue characterisation of fluorescence. Tissue samples were stored protected from light, and analysed using a fluorescence microscope. Results were correlated with histopathology. In all, 14 sentinel lymph nodes, seven axillary lymph nodes and seven primary tumours were analysed. Metastatic sentinel lymph nodes demonstrated a statistically significant higher FI than nonmetastatic sentinel lymph nodes (2630 *vs* 526, *P*<0.0001). The FI of metastatic sentinel lymph nodes, of metastatic axillary lymph nodes and of the primary tumour were comparably high, and were statistically significantly higher compared to the normal mammary tissue. Intraoperatively, only in a few cases, it was possible to recognise the metastatic sentinel lymph node macroscopically with blue light. Our study indicates that photodynamic diagnosis with ALA has a potential in the diagnosis and detection of the sentinel lymph node in patients with breast cancer, and is worth to be further investigated and developed for intraoperative photodynamic diagnosis and possibly therapy.

Photodynamic therapy (PDT) and diagnosis (PDD) is of increasing interest in oncology, especially in gynaecologic malignancies as well as in patients with breast cancer. Photodynamic therapy and PDD were introduced three decades ago, with a major focus on gynaecology (Dougherty and Marcus, 1992). Systemic or topical application of the prodrug 5-aminolevulinic acid (ALA) induces the endogenous synthesis and accumulation of large quantities of the endogenous photosensitiser protoporphyrin IX (PpIX) in many tissues, especially derivatives of epithelia such as carcinomas. Exposure to activating red light of 635 nm produces a phototoxic effect, resulting in tumour cell necrosis. Topical ALA-based PDT has been reported effective for the treatment of a variety of dermatological diseases including cutaneous superficial and nodular basal cell carcinoma, Bowen's disease and actinic (solar) keratoses (Morton *et al*, 2001). Local intrauterine application of ALA has also been used for selective endometrial ablation in animal models and in human clinical studies (Steiner *et al*, 1995,1996b; Wyss *et al*, 2001). By the use of blue light at 400 nm, PpIX induced by ALA has been applied as a fluorescence detection marker for photodiagnosis of cancer and dysplastic conditions of the urinary bladder and other organs (Kennedy *et al*, 1996). Application of ALA has been developed for detection of alterations in gynaecological tissues including the endometrium, the vulva and skin metastasis of breast cancer (Fehr *et al*, 1996,^2001^; Steiner *et al*, 1996a). Photodynamic therapy provides an alternative treatment modality using a second-generation photosensitiser and laser light to induce selective tumour necrosis (Wyss *et al*, 2001).

In a recent feasibility study applying ALA orally, we demonstrated that mammary tumour tissue examined by fluorescence microscopy presented significantly higher fluorescence intensity (FI) than the normal surrounding tissue. Autofluorescence of the normal breast tissue was very low. It was possible to visualise tumour tissue using blue light intraoperatively (Ladner *et al*, 2001a). As the minimal invasive staging of the sentinel lymph node for potential metastases is of increasing importance, we focused our consecutive study on the photodynamic detection and diagnosis of the sentinel lymph node in patients with breast cancer.

The purpose of this follow-up study was to evaluate the effectiveness of oral application of ALA for PDD of the sentinel lymph node in patients with breast cancer. Fluorescence intensity in the primary breast tumour, in the sentinel lymph nodes and in axillary lymph nodes was analysed by fluorescence microscopy.

## MATERIALS AND METHODS

### Patients

The study was performed at the Women's hospital Fontana, Chur, Switzerland, and was approved by the ethical committee of the Canton Graubuenden Chur, Switzerland (Study 18/01, October 2001). A total of 11 patients undergoing breast-conserving surgery for invasive breast cancer were included in this study after having signed the informed consent form. All patients underwent the sentinel lymph node mapping with Nanocoll® (AmershamHealth AG, Wädenswil, Switzerland) preoperatively. Intraoperatively, a blue dye (Bleu Pathenthe V, 2.5%, Guerbet, Roissy, France) was injected around the tumour to identify the sentinel lymph node. The exclusion criteria were acute porphyria, elevated liver enzymes, renal or hepatic insufficiency, intake of hypericin-containing medication and pregnancy.

### ALA and patient protection

*δ*-ALA (ASAT AG, Zug Switzerland) was administered orally at a concentration of 30 mg per kg bodyweight, solved in 50 cl of water, 3 h prior to surgery. Patients were given 8 mg Ondansetron (Zofran®, GlaxoSmithKline AG, Münchenbuchsee, Switzerland) together with *δ*-ALA. A sun blocker (Spirig®, Egerkingen, Switzerland) was applied to the face and neck, and covered with a micropigment cream, which has shown good results in light protection during photosensitisation with ALA and Foscan® (Biolitec Pharma Ltd, Edingburgh, UK) (Schwarz *et al*, 2001). In addition, patients were protected from direct light for 24 h after application of ALA.

### Tissue conservation

Immediately after excision, the malignant tumour, the sentinel lymph node as well as the other lymph nodes were protected from light in sealed bottles, and transported immediately to the Pathology Department, Cantonal Hospital Chur, Switzerland. The primary tumour tissue, the sentinel lymph node and other lymph nodes were carefully divided into two parts by a surgical blade, and were protected from light. One part underwent the regular pathological diagnostic procedures. The other part was stored and frozen in a light-protected container at −80°C. In a dark room, 10 cryosections of 10 *μ*m thickness were prepared from tumour tissue, sentinel lymph node and axillary lymph nodes. The sections were wrapped in aluminium foil, sealed in boxes protecting against drying out and photobleaching, and then again stored at −80°C. These slides were transported on dry ice to the Research Division of Gynaecology, University Hospital, Zurich. After fluorescence microscopy, the analysed sections were additionally stained with haematoxylin and eosin and microscopically analysed to obtain histological information in this part of the tissue.

### Fluorescence microscopy and image acquisition

Tissue sections were analysed by fluorescence microscopy. From every tumour and corresponding tissue, at least five sections were analysed. Fluorescence imaging was performed with a CCD camera system (photometrics, Tucson, AZ, USA) with a Kodak KAF 1600 chip coupled to a Leitz DMR/E fluorescence microscope (Leica, Glattbrugg, Switzerland). Light from a 50 mW HO mercury lamp was filtered through a 405–420 nm broad-bandpass filter to provide excitation light. A dichroic mirror reflected the excitation light onto the sample and transmitted the fluorescence emission through a 635–655 nm broad-bandpass filter onto the detector. Instrument control, image acquisition and processing were performed using the Image Pro Plus software (MediaCybernetics, Silver Spring, MA, USA). Micrographs were acquired with an integration time of 20 s. All optical settings of the microscope were kept constant for all image acquisitions, in order to be able to compare the acquired information.

### PDD during surgery

During the surgical procedure, intraoperative PDD was performed. A laparoscopic D-light system, 20133201 (Karl Storz GmbH, Tuttlingen, Germany) with a Hopkins-lens-optical system and observatory filter, analogous to the PDD-Cystoskope 27005AIA was used to illuminate the operation field and the sentinel lymph node. Using a PDD camera system, the tissue fluorescence was documented with a video system.

### Statistical analysis

For statistical analysis, Statview® for Microsoft for Windows® was used. The FI of primary breast carcinoma, metastatic and nonmetastatic sentinel lymph nodes as well as metastatic and nonmetastatic axillary lymph nodes were compared using the two-sided Student's *t*-test.

**Figure 1:**
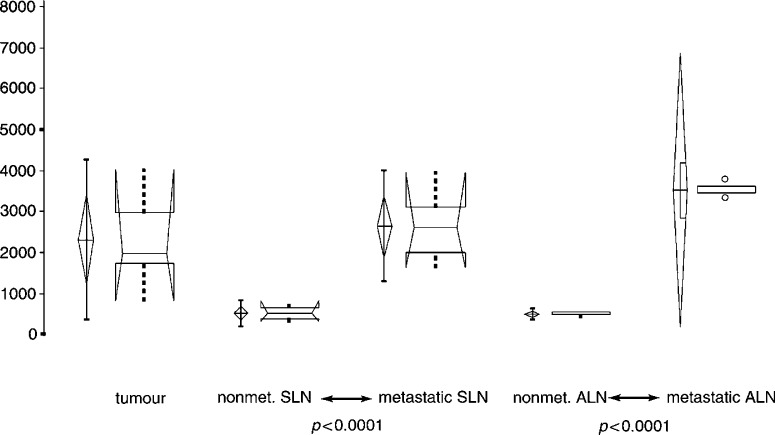
Fluorescence micrograph (× 20) of metastatic SLN (**A**) *versus* nonmetastatic SLN tissue (**B**). The FI of metastatic tissue is more intense than the FI of nonmetastatic tissue.

In the illustrations (Figure 1

), we used the Box–Whisker plots. Parametric and nonparametric summary statistics are presented for each variable. Box plots graphically show the central location and scatter/dispersion of the observations of samples. The median, the lower and upper quartiles, and confidence interval around the median are illustrated. The vertical lines show the requested nonparametric percentile ranges.

## RESULTS

### FI of the primary breast cancer compared with normal breast tissue

Sections of seven primary breast tumours were evaluated using the fluorescence microscope. The histological report of the seven primary tumours revealed six invasive ductal and one invasive lobular breast carcinoma. Tumour size ranged between 1.5 and 4.5 cm. The breast tumour tissues of patients 2, 5, 6, 8 and 11 could not be analysed due to problems of tissue conservation and technical problems with microscopic analysis. Patient 9 had bilateral breast carcinoma, and it was possible to analyse the tissue of both sides (Table 1

**Table 1:** Fluorescence intensity of tumour and lymph node tissue, correlated with histopathology

**Patient**	**Mean FI**	**Ratio**	**Histopathology**
1	832.83	6.41	Tumour
1	2590.50	19.93	Metastatic SLN
1	562.67	4.33	Nonmetastatic ALN
3	1682.33	4.99	Tumour
3	330.00	0.38	Nonmetastatic SLN
4	1771.67	12.75	Tumour
4	704.00	5.06	Nonmetastatic SLN
4	517.67	3.72	Nonmetastatic ALN
5	334.83	1.77	Nonmetastatic SLN
5	412.83	2.18	Nonmetastatic SLN
6	519.83	1.66	Nonmetastatic SLN
6	539.33	1.72	Nonmetastatic ALN
7	4029.83	12.07	Tumour
7	3921.50	11.74	Metastatic ALN
7	3954.00	11.84	Metastatic SLN
7	3341.33	10.00	Metastatic SLN
8	814.17	3.11	Nonmetastatic SLN
9	1966.17	7.28	Tumour
9	3824	14.17	Tumour
9	477.33	1.77	Nonmetastatic ALN
9	402.67	1.49	Nonmetastatic ALN
9	2072	7.67	Metastatic SLN
9	567.50	2.10	Nonmetastatic SLN
10	2106.50	9.80	Tumour
10	1640.67	7.63	Metastatic SLN
11	2852.17	11.41	Metastatic SLN
11	1958.67	7.83	Metastatic SLN
11	3300.33	13.20	Metastatic ALN

SLN=sentinel lymph node; ALN=axillary lymph node; ratio indicates the mean FI of tumour or lymph node tissue over the FI of normal breast tissue.

). All primary breast cancers showed a highly significant difference (*P*<0.0001) in FI compared to the surrounding normal mammary tissue (Figure 1). The mean value of tumour tissue was five times higher than the surrounding normal mammary tissue, and ranged from 832 to 4029 (relative values, Table 1). The normal mammary tissue ranged from 130 to 337. One patient with extensive disease (large primary tumour and three positive lymph nodes) showed the highest FI of the primary tumour (4029, patient 7, Table 1). To overcome the fact that FI varies among the patients, a ratio between the FI of tumour and normal mammary tissue was calculated and compared, yielding the same statistically significant results.

### FI of the nonmetastatic and metastatic sentinel lymph nodes and the nonmetastatic and metastatic axillary lymph nodes

Sections of a total of 21 lymph nodes were examined. In all, 14 lymph nodes were identified as sentinel lymph nodes intraoperatively (Table 2

**Table 2:** Mean values of FI of subgroups with the corresponding standard deviation, and confidence intervals

**Group**	***n***	**Mean**	**Standard deviation**	**Standard error**	**95% Confidence interval of the mean**	**Median**
Tumour	7	2316.2	1174.5	443.9	1230.0–3402.5	1966.1
Nonmetastatic SLN	7	526.2	184.5	69.7	355.5–696.8	519.8
Metastatic SLN	7	2629.9	821.7	310.6	1869.9–3389.8	2590.5
Nonmetastatic ALN	5	499.9	62.8	28.1	421.9–577.9	517.7
Metastatic ALN	2	3610.9	439.2	310.6	−335.4–7557.3	3610.9

). Histopathology diagnosed seven metastatic sentinel lymph nodes, seven disease-free sentinel lymph nodes, two metastatic axillary lymph nodes and five disease-free axillary lymph nodes (Table 1). Fluorescence intensity of the metastatic sentinel lymph nodes was significantly higher than that of nonmetastatic sentinel lymph nodes (*P*<0.0001) (Figures 1 and 2

**Figure 2:**
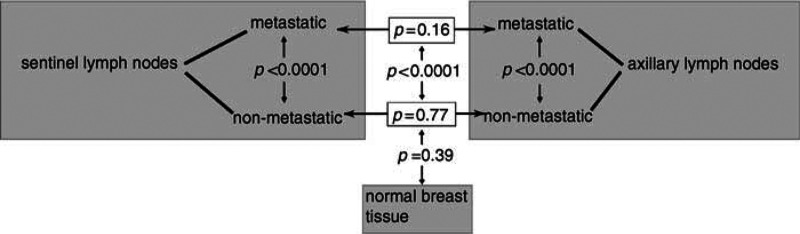
Illustration of significance levels for all groups observed. Two-sided *t*-test was used to determine significant differences. All nonmetastatic subgroups are significantly different from the tumour tissue as well as metastatic lymph nodes.

). Fluorescence intensity of metastatic axillary lymph nodes was also significantly higher than that of nonmetastatic axillary lymph nodes (*P*<0.0001) (Figures 1 and 2). Metastatic axillary and metastatic sentinel lymph nodes did not show a significant difference in FI (Figures 1 and 2). Also, nonmetastatic sentinel lymph nodes and nonmetastatic axillary lymph nodes did not differ in FI (Figure 2). Figure 3

**Figure 3:**
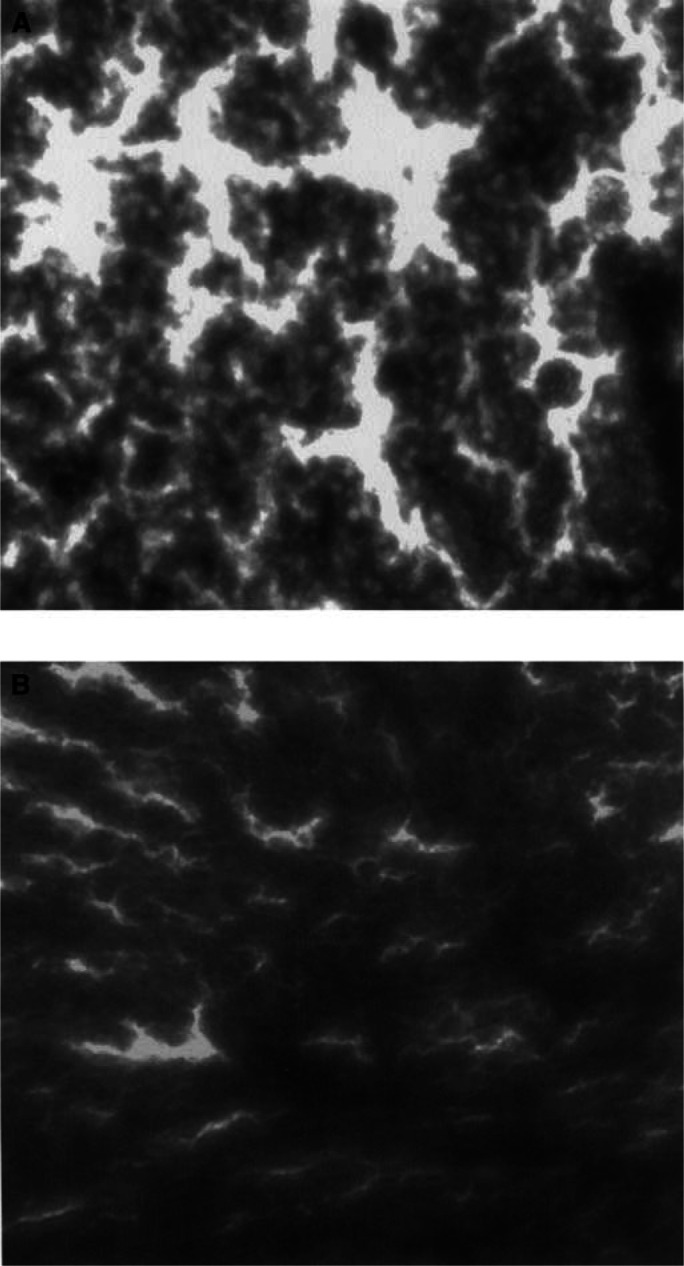
Box–Whisker plot of tumour and lymph node groups. Graphs on the left side illustrate parametric, on the right side nonparametric observations.

gives an example for the microscopical findings of a metastatic and a nonmetastatic sentinel lymph node.

### FI of the primary breast cancer compared with metastatic and non-metastatic lymph node tissue

FI of the primary breast cancer tissue and metastatic lymph node tissue was comparably high. Fluorescence intensity of primary breast cancer tissue was significantly higher than the one of the nonmetastatic lymph node tissue (*P*<0.0001) (Figure 1).

### Intraoperative fluorescence of tumour and lymph nodes

Only a limited evaluation of the macroscopic fluorescence was possible using the laparoscopic D-light system intraoperatively. Six sentinel lymph nodes showed fluorescence intraoperatively. In three of the six lymph nodes, metastatic tumour tissue was found by histopathology. Seven nonmetastatic lymph nodes did not show any fluorescence. Five of a total of 11 tumours showed fluorescence intraoperatively. The tumour cavity in all patients was lightened during the operation and did not show any fluorescence, as the tumour tissue was excised with negative margins at histopathology at the first intent.

### Side effects from oral application of ALA

Oral application of ALA at a concentration up to 60 mg per kg bodyweight is known to have little and short-lived side effects (24 h). We used a concentration of 30 mg per kg bodyweight. Applying this concentration and all the safety procedures, none of the 11 patients developed notable side effects. Two patients had slightly elevated liver enzymes postoperatively, none of the patients had nasal swelling, oedema or erythema of the skin.

## DISCUSSION

Lymphatic mapping and identification of the sentinel lymph node in patients with breast cancer is rapidly approaching the standard of care. The driving forces behind this rapid acceptance are mainly a result of the accuracy of the procedure with a significant decrease in morbidity over the old standard of care, a level I and II node dissection (Jakub *et al*, 2003). It is of increasing interest to quickly detect the sentinel lymph node and to know whether there it is metastasised in order to decide whether axillary lymph node dissection becomes necessary. Our initial set-up for PDD after orally applied ALA (Ladner *et al*, 2001a) was successfully confirmed in this follow-up study for the demonstration of FI in tumour tissue. This was possible with an even lower ALA concentration of 30 mg kg^−1^ bodyweight. Fluorescence intensity of all tumour samples was significantly higher than FI of the surrounding normal mammary tissue, and autofluorescence of normal tissue was very low. These findings confirm our earlier results. One limitation of our study is the small patient population of 11 included patients. Nevertheless, with the analysis of 21 lymph nodes it was possible to yield statistically significant results.

In this study, we found significantly higher FI in metastatic sentinel lymph nodes as well as in other metastatic axillary lymph nodes compared to the normal breast tissue and to nonmetastatic sentinel and axillary lymph node tissue. Intraoperatively, three metastatic lymph nodes and five of the primary breast tumours showed fluorescence using a laparoscopic D-light system. As the laparoscopic D-light system is usually not used for open surgery, it was difficult to assess the macroscopic fluorescence adequately. Based on our results, we believe that it is worth emphasising on the development of an optimal light system to detect the fluorescence of the sentinel lymph node in the axilla. With improved sensor technology (Wagnieres *et al*, 1998; Bigio *et al*, 2000), it might become possible to use ALA-based PDD to reliably identify the axillary sentinel lymph node in patients with breast cancer. Even intraoperative PDT may be envisioned once the sentinel lymph node has been identified by PDD carrying tumour metastasis. This would offer new minimal invasive treatment modalities after diagnosing the extent of disease.

We found a large variation in FI among our patients. It seems notable that, in patients with more extensive disease (Table 1, patient 7 and patient 11), FI was considerably higher than in patients with local disease. Many factors influence the amount of PpIX accumulating in the tissue. Metabolism of ALA varies from one patient to another. With a higher turnover of cells, FI might increase.

Various ways to apply different photosensitisers have been studied. Photodynamic treatment of local chest wall recurrence of breast cancer offered a minimal-invasive, outpatient treatment modality, with few side effects but prolonged healing phase (Wyss *et al*, 2001). 5-Aminolevulinic acid is known to produce side effects such as nausea and phototoxic reaction of the light-exposed skin (Webber *et al*, 1997; Ladner *et al*, 2001b). Up to 60 mg per kg bodyweight, ALA is associated with minimal side effects in patients undergoing surgery. Based on the results of the recent study performed at our institution in which pranasal swelling was a common finding (Ladner *et al*, 2001a), we decided to administer ALA at a dosage of 30 mg per kg bodyweight. With this dosage, we had no notable side effects and only in two patients transient elevated liver enzymes were observed. One might hypothesise that a higher dosage would result in easier macroscopic detection of tumour tissue or the sentinel lymph node with the D-light system. However, microscopic FI of the tissue was measurable without problems in the cases reported in this study.

Our study indicates that PDD with ALA has a potential in the detection and diagnosis of the diseased sentinel lymph node in patients with breast cancer. This technique is worth to be further investigated and developed for intraoperative PDD and possible therapy.
